# Population pharmacokinetics of nalbuphine in patients undergoing general anesthesia surgery

**DOI:** 10.3389/fphar.2023.1130287

**Published:** 2023-03-21

**Authors:** Xuyang Nie, Xiaonan Gao, Jinglin Gao, Tianfang Heng, Yuqi Zhang, Yaqi Sun, Zhangying Feng, Li Jia, Mingxia Wang

**Affiliations:** ^1^ Department of Clinical Pharmacology, The Fourth Hospital of Hebei Medical University, Shijiazhuang, China; ^2^ Department of Anesthesiology, The Fourth Hospital of Hebei Medical University, Shijiazhuang, China

**Keywords:** Nalbuphine, Population pharmacokinetics, NONMEM, induced anesthesia, general anesthesia surgery

## Abstract

**Purpose:** The aim of this study was to build a population pharmacokinetics (PopPK) model of nalbuphine and to estimate the suitability of bodyweight or fixed dosage regimen.

**Method:** Adult patients who were undergoing general anesthetic surgery using nalbuphine for induction of anesthesia were included. Plasma concentrations and covariates information were analyzed by non-linear mixed-effects modeling approach. Goodness-of-fit (GOF), non-parametric bootstrap, visual predictive check (VPC) and external evaluation were applied for the final PopPK model evaluation. Monte Carlo simulation was conducted to assess impact of covariates and dosage regimens on the plasma concentration to nalbuphine.

**Results:** 47 patients aged 21–78 years with a body weight of 48–86 kg were included in the study. Among them, liver resection accounted for 14.8%, cholecystectomy for 12.8%, pancreatic resection for 36.2% and other surgeries for 36.2%. 353 samples from 27 patients were enrolled in model building group; 100 samples from 20 patients were enrolled in external validation group. The results of model evaluation showed that the pharmacokinetics of nalbuphine was adequately described by a two-compartment model. The hourly net fluid volume infused (HNF) was identified as a significant covariate about the intercompartmental clearance (Q) of nalbuphine with objective function value (OFV) decreasing by 9.643 (*p* < 0.005, *df* = 1). Simulation results demonstrated no need to adjust dosage based on HNF, and the biases of two dosage methods were less than 6%. The fixed dosage regimen had lower PK variability than the bodyweight regimen.

**Conclusion:** A two-compartment PopPK model adequately described the concentration profile of nalbuphine intravenous injection for anesthesia induction. While HNF can affect the Q of nalbuphine, the magnitude of the effect was limited. Dosage adjustment based on HNF was not recommended. Furthermore, fixed dosage regimen might be better than body weight dosage regimen.

## 1 Introduction

Surgery is an essential component of global healthcare. In 2008, researchers found that approximately 234.2 million major operations were performed annually (Weiser et al., 2008). In clinic, patients are usually given a combination of sedatives and opioid analgesics for the induction of intravenous anesthesia before surgery. However, analgesic drugs commonly used in anesthesia induction, such as fentanyl, sufentanil and remifentanil, are often accompanied by adverse reactions such as cough, dizziness, respiratory depression and hemodynamic instability (Hirsch et al., 2015; Shuying et al., 2016).

As a semi-synthetic opioid analgesic with analgesic potency comparable to morphine, nalbuphine is primarily used to prevent and treat moderate to severe pain, including pain treatment before to surgery, following surgery, and during childbirth (Errick and Heel, 1983). Notably, unlike other opioids, nalbuphine can not only activate κ opioid receptors, but also partially antagonize μ opioid receptors (Schmidt et al., 1985). Due to this distinct hybrid agonist-antagonist opioid receptor activity, nalbuphine provides analgesia with fewer side effects. Firstly, nalbuphine has a “capping effect” on respiratory inhibition when the dose exceeds 0.3–0.5 mg/kg (Giannina et al., 1995). Secondly, after the use of nalbuphine in general anesthesia, patients were hemodynamically stable during surgery, recovered consciousness rapidly after surgery, and had a low incidence of nausea, vomiting and emergence agitation (Sear et al., 1987; Dalens et al., 2006; Chawda et al., 2010; Kubica-Cielinska and Zielinska, 2015). Additionally, nalbuphine is efficient in reducing propofol dosage and the pain associated with its injection, and in reducing the sensitivity of postoperative pain caused by high doses of remifentanil or sufentanil (He et al., 2021). Thus, nalbuphine also has been widely used in the induction and maintenance of general anesthesia.

Nalbuphine has been used clinically for more than 40 years, and its pharmacokinetics (PK) had been extensively studied in infants, children, healthy adult and elderly patients, but remain limited in patients undergoing general anesthesia surgery. Sear et al. (1987) found that nalbuphine exhibited lower total clearance (CL) and apparent volume of distribution (V_d_) in anesthetized patients compared with the results obtained in awake volunteers. Similarly, our previous study (Gao et al., 2022) found that nalbuphine CL(33.42 L/h) and V_d_ (137.69 L) were significantly lower in patients undergoing general anesthesia than in healthy patients (CL 90.0 L/h; V_d_ 326.5 L) (He et al., 2021), regardless of whether the patient had liver dysfunction. However, neither of these studies analyzed the reasons for the above-mentioned results. Thus, it is necessary to further explore the factors that affect the PK of nalbuphine during general anesthesia surgery.

In addition, the choice of nalbuphine dosage regimens is also an issue worth to be discussed. Wang et al. (2022) found that the PK variability of the drug was lower for fixed-dose dosage regimens than that for weight-based dosage regimens. Previous studies had shown that weight of neonates and children is a significant factor affecting nalbuphine PK behavior (Jacqz-Aigrain et al., 2003; Bressolle et al., 2011). In China, nalbuphine is also recommended to be administered by body weight (0.2 mg/kg) for induction of anesthesia in clinic and drug package insert. However, nalbuphine is often administered at a fixed dose (10–20 mg) in many PK studies in adults (Sear et al., 1987; He et al., 2021). Thus, it also needs to be considered whether it is necessary to administer nalbuphine by weight when used for induction of anesthesia.

Population pharmacokinetics (Pop PK) analysis is a valid and scientific method for describing PK behavior and identifying sources of variability, and that plays an increasing role in clinical drug studies. Although PopPK of nalbuphine has been reported in studies from neonates to children undergoing general anesthesia and post-surgery (Bressolle et al., 2011; Pfiffner et al., 2022), adult patients undergoing general anesthesia surgery has not been included in their studies. Therefore, the purpose of this study was to analyze the factors affecting the PK of nalbuphine in adult patients undergoing general anesthesia using PopPK analysis method, and to perform dosage regimen simulations to evaluate and optimize the clinical dosing strategy for this study population.

## 2 Materials and methods

### 2.1 Study design

Participants were patients who scheduled for elective surgery at the Fourth Hospital of Hebei Medical University in 2021. Exclusion criteria were 1) allergic to nalbuphine, 2) contraindications to general anesthesia, 3) III and IV grades of intubation according to the Mallampati classification, 4) a history of asthma, chronic pain or chronic cough, 5) known or suspected cardiopulmonary, renal or metabolic disease, 6) pregnant, 7) long-term opioid medications, and 8) excessive intraoperative bleeding. A total of 47 patients were recruited in this research. Among them, twenty-seven patients who received intensive PK sampling were used for build model. The remaining 20 patients were used for external verification of PopPK model.

Before operation, all patients were routinely fasted overnight, and did not use sedative or analgesic drugs within 24 h. After entering the operating room, the electrocardiogram (ECG), heart rate (HR), saturation of pulse oximetry (SpQ2) and bispectral index (BIS) of patients were continuously monitored. Then radial artery puncture was performed under local anesthesia and was used to monitor the mean arterial pressure of patients. The left and peripheral vein were opened for drug injection and blood collection, respectively. Anesthesia induction was performed after preoxygenation 5 min with 100% oxygen. Nalbuphine (Yichang Human well Pharmaceutical, Hubei, China) 15 mg was injected over 2–3 min, followed by 0.05 mg/kg midazolam, 0.2 μg/kg sufentanil, 0.03 mg/kg etomidate, and cisatracurium 0.2 mg/kg. After tracheal intubation, anesthesia was maintained with sevoflurane and remifentanil, and intermittent injections of cis-atracurium were used to maintain inotropy.

This study was conducted in accordance with principles in the Declaration of Helsinki, and was approved by the ethics committee of the Fourth Hospital of Hebei Medical University, Shijiazhuang, China (No. 2019121). Informed consent was obtained from all participants (Gao et al., 2022).

### 2.2 Sampling and bioanalytical methods

Blood samples (2 mL) were drawn: before dosing and at 3, 5, 10, 15, 30 and 45 min and 1, 1.5, 2, 3, 4, 5, 6 and 12 h after nalbuphine administration for modelbuilding group; before dosing, during endotracheal intubation and at 1, 3 and 10 min after endotracheal intubation for external validation group.

The blood samples were heparinized and centrifuged, with plasma samples stored at −80°C until analysis. Plasma nalbuphine concentrations were measured using a validated ultra-performance liquid chromatography-tandem mass spectrometry (UPLC-MS/MS) method after protein precipitation with acetonitrile. The lower limit of quantitation (LLOQ) was 0.1 ng/mL. The calibration range was 0.1–500 ng/mL (Gao et al., 2022).

### 2.3 Population pharmacokinetic model development

Non-linear mixed effect modelling was performed by NONMEM^®^ (version 7.5.0) interfaced by MaS studio (version 1.6.0.5) and Perl-speaks-NONMEM (PsN, version 5.2.6) toolkit. Statistical analyses and graphical visualizations of NONMEM output post-processing were carried out with RStudio (version 1.2.5033) using R software (version 4.2.1).

#### 2.3.1 Basic structure model

Based on graphical exploratory analysis, the plasma concentrations of nalbuphine were modeled by one- and two-compartment models using first-order conditional estimation with the η–ε interaction (FOCE-I) method. Between-subject variability (BSV), as a structural pharmacokinetic parameter, was assumed to be log-normally distributed and was applied by exponential model. For estimating the residual unexplained variability (RUV), three models were tested, including proportional, additive and a combination of a proportional error model and an additive error model.

The optimal structural model selection was based upon objective function value (OFV), precision of parameter estimates and visual inspection of goodness-of-fit (GOF) plots for nested models, and Akaike’s Information Criterion (AIC) for non-nested models.

#### 2.3.2 Covariate model

The demographic statistics information, disease information and clinical laboratory measurements were collected as potential covariates. Only the covariates with missing values less than 20% of patients were included in covariate evaluation.

The correlation between covariates and covariates, covariates and PK parameters were firstly investigated using statistical and graphical method. When correlations existed between covariates (correlation coefficient > 0.7), only one of the covariates more commonly used clinically could be included in subsequent analyses to avoid multicollinearity and instability of parameter estimates. A stepwise forward inclusion and backward elimination process were then tested formally.

In forward inclusion process, covariates were added individually to the basic model one by one. Continuous covariates were tested by a linear function (Eq. 1), a power function (Eq. 2), or an exponential function (Eq. 3), while categorical covariates were assessed by Eq. 4. And the covariate would be included in the basic model if the OFV decrease more than 3.84 (*p* < 0.05, *df* = 1). All covariates that individually had a potential influence on the basic model were available after the first step of inclusion. The model with most decreasing OFV could be used as the base covariate model for subsequent analysis. Then each of potentially influencing covariates was added to the base covariate model and a decrease of OFV exceeding 6.63 was considered to be significant (*p* < 0.01, *df* = 1). A full model was constructed when all significant covariates were incorporated into the basic model. In backward elimination process, covariates from the full model were removed one at a time. An increase of OFV > 7.88 (*p* < 0.005, *df* = 1) was considered as a criterion to retain a covariate in model. The final model was formed when basic model combined with all covariates that met the above statistical criteria and had an impact over 20% on the parameter.

$$\theta_{i}=\theta_{1}+\theta_{2}\cdot \left({cov}_{i}/{cov}_{median}\right)$$ (1)

$$\theta_{i}=\theta_{1}\cdot {\left({cov}_{i}/{cov}_{median}\right)}^{\theta_{2}}$$ (2)

$$\theta_{i}=\theta_{1}\cdot {\theta_{2}}^{\left({cov}_{i}/{cov}_{median}\right)}$$ (3)

$$\theta_{i}=\theta_{1}\cdot {\theta_{2}}^{{cov}_{i}}$$ (4)

where *θ*_*i*_ describe the pharmacokinetic parameter value for the individual i; *θ*_1_, and *θ*_2_ describe the typical value of a pharmacokinetic parameter; *cov*_i_ and *cov*_median_ describe covariate values for the *i*th individual and the population median, respectively.

#### 2.3.3 Model evaluation

GOF, non-parametric bootstrap and visual predictive check (VPC) were applied for internal model evaluation (Nguyen et al., 2017).

GOF plots were used to evaluated model fitness, including prediction-based diagnostic plots [observation versus population prediction (PRED), observation versus individual prediction (IPRED)] and residual-based model diagnostic plots (conditional weighted (CWRES) versus PRED, CWRES versus time). When data points were randomly distributed near y = x in the prediction-based diagnostic plots and most of the CWRES were within ± 2 in the residual-based model diagnostic plots, and the locally weighted regression (LOESS) lines did not show obvious bias, suggesting that the model well described the trend of data concentration. Note that the DV-IPRED diagnostic map can be applied as a basis for model evaluation only when the contraction value of the residual variance was less than 30%.

Non-parametric bootstrap was performed for final model with 1,000 times resampling. The median and the 2.5th and 97.5th percentiles of the PopPK parameter estimates from successfully minimized resampled datasets were compared with the final model parameter estimates. If the 95% confidence interval for each parameter contains the final model parameters and the proportion of successful model minimization (robustness rate) > 80%, the model is stable and the parameters have high confidence.

In order to adjust for differences of independent variables, a prediction corrected visual predictive check (pcVPC) method was conducted by simulating 1,000 datasets based on the final model, and used to evaluate the variability and central tendency between the observed and simulated data. The median and 5th and 95th percentiles of the prediction corrected simulated data were compared with that of the prediction corrected observed data. When the number of observations falling outside the simulation-based 90% prediction interval was less than 10%, the model could be considered to have a high prediction accuracy.

In addition, the final model was externally evaluated using the following two datasets: external evaluation dataset without observations and external evaluation dataset within the first observation of each subject. The relative prediction error (PE%), the median prediction error (MDPE), the median absolute prediction error (MAPE) and the percentage of PE% falling within the ± 20% and ± 30% were calculated to reflect accuracy and precision of the final model. The model was considered to be predictive and clinically acceptable when the standards of MDPE ≤ ± 20%, MAPE ≤ ± 30%, F_20_ ≥ 35% and F_30_ ≥ 50% were reached.

#### 2.3.4 Simulations

##### 2.3.4.1 The evaluation of the effect of covariates on the PK of nalbuphine

Based on the final model, a Monte Carlo simulation approach was used to evaluate the effect of different levels of covariates on the PK of nalbuphine following 15 mg or 0.2 mg/kg intravenous injection once only. The various covariates levels were set as 10th, 50th and 90th percentile of the model building population. The Monte Carlo simulation was run 1,000 times at each covariate level.

##### 2.3.4.2 Dosage regimen simulation and statistical analysis

In this study, fixed dose regimen (15 mg) was explored in patients undergoing anesthesia surgery based on published literature (Cai et al., 2011; He et al., 2021). To assess the appropriateness of fixed dosage or bodyweight dosage strategies on the PK of nalbuphine in patients undergoing general anesthesia, two dosing regimens, 12 mg and 0.2 mg/kg, were selected for the study based on the drug instructions.

Based on the final model, PK profiles of two dose regimens were simulated for 1,000 times, respectively, applying the weight and the HNF information of 27 patients from the model-building dataset. The sampling time was set at pre-dose and 0.05, 0.08, 0.17, 0.25, 0.5, 0.75, 1, 2, 3, 4, 6, 8, 10 and 12 h after dosing. Statistical analyses were used to compare the concentration at the 0.05 h, 12 h and median operation time of fixed dosage regimen with that of bodyweight dosage regimen; Scatter plots were used to show the PK profiles of nalbuphine of two dosage regimens.

### 2.4 The effectiveness and safety outcomes of patients

Hemodynamic indices and sedation levels were recorded for all patients around the administration of nalbuphine, around tracheal intubation and intraoperatively; adverse effects such as respiratory depression, agitation, nausea and vomiting were recorded for patients intraoperatively and postoperatively.

## 3 Results

### 3.1 Patients and datasets

Blood samples from patients used for the model building group were collected according to the study design. Blood samples from patients used for the external validation group were collected by our anesthesiology researcher at a later stage of the model construction according to the same dosing protocol. Due to the difficulty of obtaining clinical samples and in the principle of maximizing the use of clinical sample resources, this data was obtained after communication with the anesthesiology researchers. Because of its sparse sampling sites, it was only used for external validation. In this study, a total of 458 plasma concentrations were collected from 48 patients. The concentrations of all samples except pre-dose time were more than the LLOQ. And no data were recognized as outliers. However, one subject with 5 samples was excluded due to the lack of HNF in external validation group. In total, there were 27 patients with 353 samples and 20 patients with 100 samples in model building group and external validation group, respectively.

**FIGURE 1:**
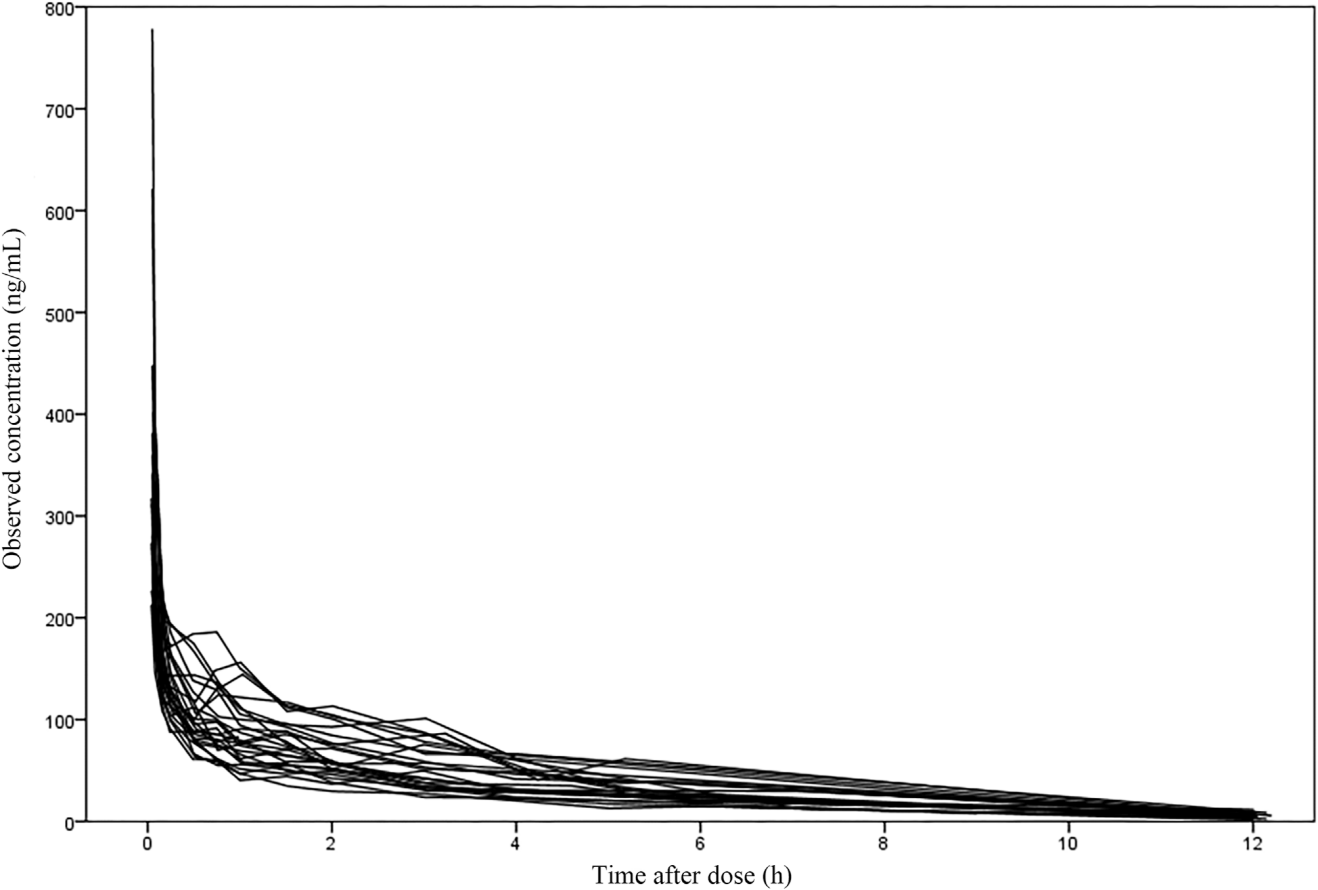
The concentration-time profile of nalbuphine in the building group (27 subjects with 353 samples).

The 47 patients enrolled were aged 21–78 years and weighed 48–86 kg. 29.8% of patients underwent lumpectomy and 70.2% underwent open surgery. Among them, liver resection accounted for 14.8%, cholecystectomy for 12.8%, pancreatic resection for 36.2% and other surgeries for 36.2%. The demographic statistics information, disease information and clinical laboratory measurements of two groups were listed in Table 1. The concentration versus time profile of building group was displayed in Figure 1.

**TABLE 1:** Baseline demographics characteristics and clinical laboratory measurements of patients included in the analysis.

	Model-building dataset	External validation dataset
Number of patients	27	20
Number of concentrations	353	100
Dose (mg/kg)	0.24 ± 0.04, (0.24, 0.18–0.31)	0.24 ± 0.05, (0.2, 0.18–0.32)
Age (years)	53.85 ± 16.63, (58, 21–76)	52.5 ± 13.56, (54.5, 27–78)
Height (cm)	163.96 ± 6.87, (165, 153–175)	159.85 ± 8.05, (157.5, 148–175)
Total body weight (kg)	63.32 ± 9.3, (62, 48–82)	62.25 ± 9.67, (59.5, 50.8–86)
Body mass index (kg/m^2^)	23.54 ± 3, (23.23, 16.61–30.12)	24.2 ± 2.77, (23.28, 20.8–29.9)
Systolic pressure (mmHg)	138.8 ± 17.22, (138, 102–170)	135.21 ± 13.83, (138, 117–160)
Diastolic pressure (mmHg)	77.26 ± 9.27, (78, 60–92)	75 ± 10.06, (75.5, 60–92)
Heart rate (/min)	71.37 ± 14.48, (70, 50–110)	83.05 ± 9.83, (79, 70–100)
Alanine aminotransferase (U/L)	58.86 ± 69.62, (23.6, 6–260.9)	35.37 ± 52.46, (15.45, 5.5–200.9)
Aspartate aminotransferase (U/L)	42.16 ± 33.64, (24.4, 10.6–128.7)	28.4 ± 27.28, (18.75, 9.6–113.7)
Gamma-glutamyl transferase (U/L)	191.87 ± 319.87, (77.2, 8.4–1,493.2)	129.93 ± 286.12, (18, 8.4–1,219.1)
Total protein (g/L)	66.79 ± 8.67, (66.3, 50–82)	70.95 ± 5.8, (72, 58.5–78.9)
Albumin (g/L)	40.29 ± 5.63, (40.8, 29.2–50.7)	40.73 ± 5.03, (42.1, 29.5–48.1)
Globulin (g/L)	26.51 ± 4.84, (26.4, 15–35.9)	30.22 ± 3.49, (30.1, 24.1–38.8)
Total bilirubin (μmol/L)	25.83 ± 28.65, (13.18, 3.7–104.34)	26.47 ± 40.57, (10.07, 5.51–169.9)
Serum creatinine (μmol/L)	56.69 ± 14.47, (55.9, 34–90.1)	48.92 ± 15.44, (48.85, 23.6–98.9)
Creatinine clearance (mL/min)	111.19 ± 27.38, (105.14, 53.4–160.98)	130.38 ± 45.6, (124.01, 65.72–263.83)
Uric acid (μmol/L)	281.46 ± 90.59, (297.5, 124.4–463.3)	259.11 ± 80.71, (247.7, 152.1–428.9)
Hemoglobin (g/L)	123.83 ± 22.03, (124, 87–178)	119.94 ± 25.88, (122.5, 71–162)
D dimer (mg/L)	0.27 ± 0.42, (0.1, 0.02–2.02)	0.34 ± 0.63, (0.16, 0.03–2.57)
Hourly net fluid volume infused (mL/h)	617.96 ± 247.61, (563.56, 234.26–1,202.25)	634.19 ± 178.94, (610.42, 314.29–1,047.24)
Operation duration (h)	4.31 ± 1.9, (4, 0.83–8.33)	3.59 ± 1.78, (3.05, 1.27–7.85)
Female	15 (55.6%)	14 (70%)
Smoke	11 (40.7%)	2 (10%)
Drink	11 (40.7%)	1 (5%)
Tumor	21 (77.8%)	17 (85%)
Hepatobiliary disease	15 (55.6%)	7 (35%)
Hypertension	7 (25.9%)	6 (30%)
CTP-B	5 (18.5%)	4 (20%)
Laparoscopic surgery	8 (29.6%)	6 (30%)

Data were expressed as mean ± standard deviation (median, range) for continuous covariates, and number (percentage) for categorical covariates. Creatinine clearance was calculated with Cockcroft-Gault formula for male as followed: ${CL}_{Cr}\left(mL\cdot \min^{-1}\right)=\left(140-AGE\right)\times WT\left(kg\right)÷\left(S_{Cr}\left(\mu mol\cdot L^{-1}\right)\times 0.81\right)$ Creatinine clearance in female = male value × 0.85. Hourly net fluid volume infused (HNF) was calculated with the following formula: $HNF=\left(FVI+BVI-UVO\right)÷OT$ where FVI, BVI, and UVO, was fluid volume infused, blood volume infused, and urine volume output during surgery, respectively. OT, was operation time. CTP-B: Number of patients with a liver function class of B according to Child-Turcotte-Pugh (CTP) classification; the others were class A.

### 3.2 Population pharmacokinetic model

**FIGURE 2:**
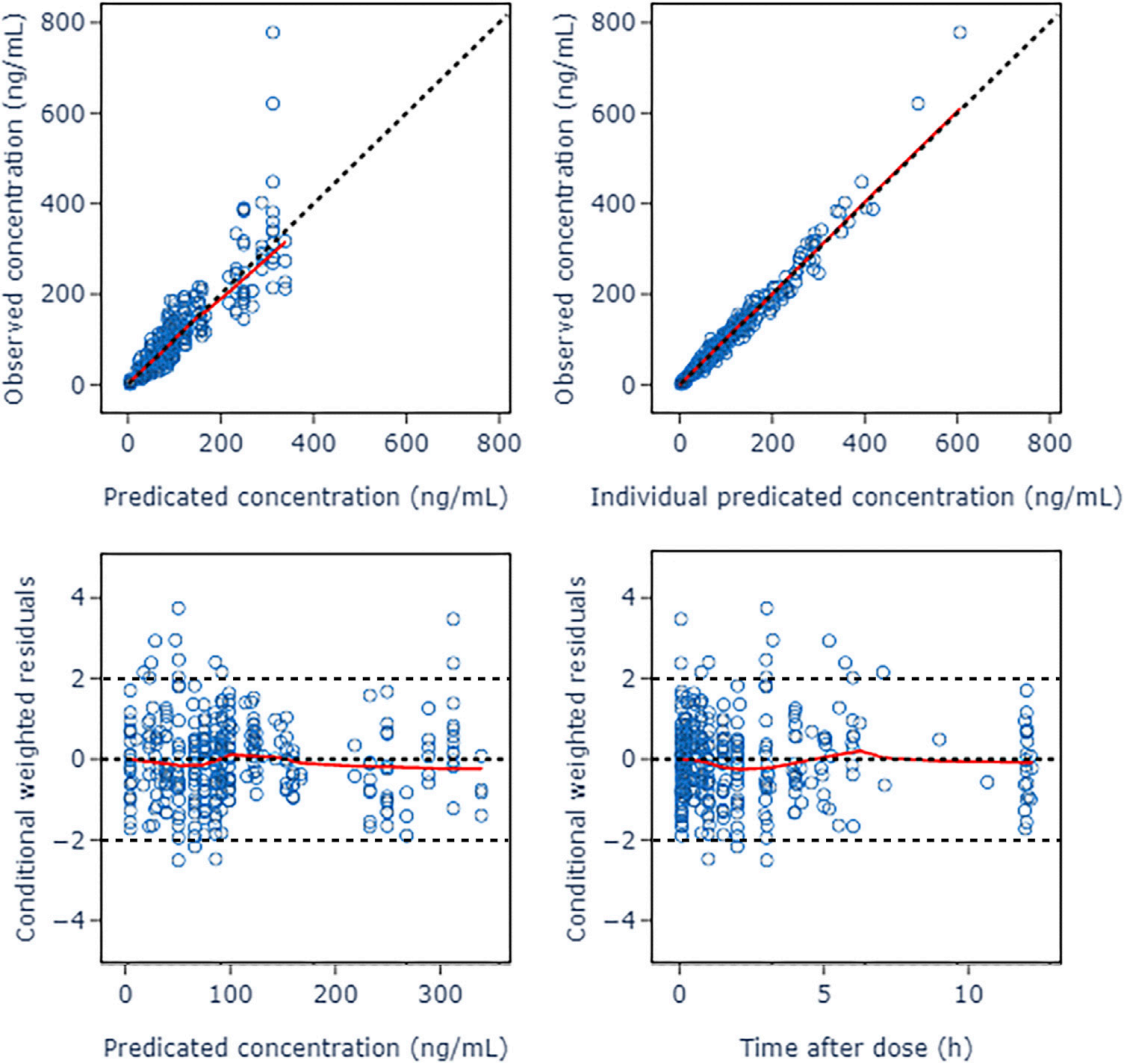
Goodness of fit plots of nalbuphine basic PopPK model. The red lines represent the locally weighted scatterplot with smoothing.

A two-compartment model was selected as the basic structure model based on results from the model building dataset and previously published data. Compared with one-compartment model, it decreased AIC by 519.209, and had a good GOF diagnosis plot (Figure 2). The model was parameterized by CL, volume of distribution for the central compartment (V_1_), intercompartmental clearance (Q) and volume of distribution for the peripheral compartment (V_2_). The BSV was incorporated into all parameters using exponential model. The RUV was described by a combination of a proportional error model and an additive error model. The Population pharmacokinetic parameter estimates of nalbuphine of the basic model were shown in Table 2.

**TABLE 2:** Population pharmacokinetic parameter estimates of nalbuphine of the basic model.

Parameter	Estimates	RSE (%)	SHRINKSD (%)
Structural model parameter			
CL	32.9	5.5	—
V_1_	31	9.9	—
Q	261	8.1	—
V_2_	85.9	7.1	—
Between-subject variability			
ωCL (%CV)	27.7	12.4	4.2
ωV_1_ (%CV)	38.9	24.2	17.0
ωQ (%CV)	35.5	17.2	10.9
ωV_2_ (%CV)	36.5	11.7	4.5
Residual unexplained variability			
Proportional error (%CV)	13.7	8.0	13.8
Additive error (ng/mL)	2.90	20.5	13.8

RES: relative standard error; CL: total clearance (L/h); V_1_: volume of distribution for the central compartment (L); Q: intercompartmental clearance (L/h); V_2_: volume of distribution for the peripheral compartment (L); ω_CL_, ω_V1_, ω_Q_ and ω_v2_: the estimates of between-subject variability of CL, V_1_, Q and V_2_, respectively.

**FIGURE 3:**
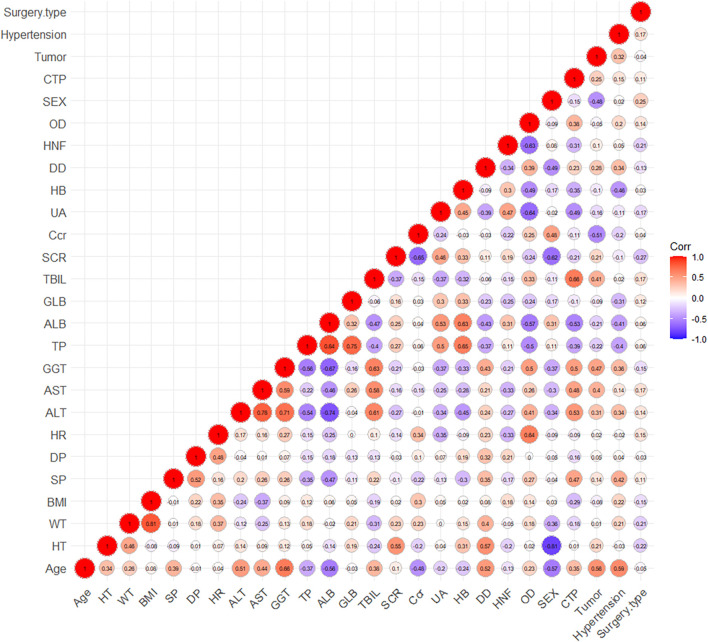
The graph of correlation between covariates.

After correlation analysis, strong correlations (R > 0.7) were found between sex and height; weight and body mass index (BMI); Aspartate aminotransferase (AST), Albumin (ALB), Gamma-glutamyl transferase (GGT) and alanine aminotransferase (ALT); ALB, Globulin (GLB) and total protein (TP) (Figure 3). These strongly correlated covariates were avoided to be included in the model at the same time. During the forward inclusion process, ALT and hourly net fluid volume infused (HNF) were identified to be a significant covariate on CL and Q, respectively. However, only HNF was remained in model with OFV decreasing by 9.643 in the backward elimination process. The stepwise process of building the PopPK model of nalbuphine were shown in Table 3. Parameter estimates of the final model were shown in Table 4 and the equations were as below:

$$CL\;\left(L/h\right)=32.9$$

$$V1\;\left(L\right)=32.5$$

$$Q\;\left(L\right)=245\cdot {\left(HNF/617.96\right)}^{-0.58}$$

$$V2\;\left(L\right)=83.5$$

**TABLE 3:** The stepwise process of building the PopPK model of nalbuphine.

Model no.	Model description	Functional expressions	OFV	ΔOFV	Compare to	*p*-value
1	Base Two-compartment model	—	2,158.168	—	—	—
Forward inclusion						
2	Add ALT on CL in model 1	a	2,150.776	−7.39	model 1	< 0.05
3	Add GGT on CL in model 1	b	2,153.271	−4.90	model 1	< 0.05
4	Add CTP on CL in model 1	c	2,153.111	−5.06	model 1	< 0.05
5	Add HR on V_1_ in model 1	b	2,151.111	−7.06	model 1	< 0.05
6	Add NFI on V_1_ in model 1	b	2,154.207	−3.96	model 1	< 0.05
7	Add OD on V_1_ in model 1	b	2,150.612	−7.56	model 1	< 0.05
8	Add HR on Q in model 1	b	2,152.292	−5.88	model 1	< 0.05
9	Add NFI on Q in model 1	b	2,148.529	−9.64	model 1	< 0.05
10	Add OD on Q in model 1	b	2,150.299	−7.87	model 1	< 0.05
11	Add Cancer on Q in model 1	c	2,152.809	−5.36	model 1	< 0.05
12	Add WT on V_2_ in model 1	b	2,154.098	−4.07	model 1	< 0.05
13	Add GGT on V_2_ in model 1	b	2,151.149	−7.02	model 1	< 0.05
14	Add UA on V_2_ in model 1	b	2,152.796	−5.37	model 1	< 0.05
15	Add DD on V_2_ in model 1	b	2,153.374	−4.79	model 1	< 0.05
16	Add NFI on V_2_ in model 1	b	2,154.225	−3.94	model 1	< 0.05
17	Add OD on V_2_ in model 1	b	2,153.697	−4.47	model 1	< 0.05
18	Add CTP on V_2_ in model 1	c	2,153.759	−4.41	model 1	< 0.05
19	Add ST on V_2_ in model 1	c	2,154.225	−3.94	model 1	< 0.05
20	Add ALT on CL in model 9	a	2,141.133	−7.40	model 9	< 0.01
Backward elimination						
21	remove NFI on Q from model 20	—	2,150.776	9.64	model 20	< 0.005
22	remove ALT on CL from model 20	—	2,148.529	7.40	model 20	> 0.005

a: linear function; b: power function; c: exponential function.

**TABLE 4:** Population pharmacokinetic parameter estimates of nalbuphine in the final model and bootstrap evaluation.

Parameter	Final model			Bootstrap			Relative bias (%)
	Estimates	RSE (%)	SHRINKSD (%)	Median	2.5th-97.5th percentile		
Structural model parameter							
CL	32.9	5.47	NA	32.8	29.46	36.54	−0.30
V_1_	32.5	10.25	NA	31.9	25.90	38.10	−1.85
Q	245	13.99	NA	247	216.63	297.38	0.82
V_2_	83.5	7.94	NA	84.6	70.70	97.34	1.32
HNF on Q	−0.58	14.11	NA	−0.557	−0.802	−0.084	−3.97
Between-subject variability							
ωCL (%CV)	27.7	12.26	4.30	26.9	19.8	33.5	−2.89
ωV_1_ (%CV)	40.1	22.92	13.10	37.4	19.8	56.4	−6.73
ωQ (%CV)	17.9	42.65	30.72	16.6	0.2	39.0	−7.26
ωV_2_ (%CV)	40.8	13.84	3.18	38.5	27.2	50.7	−5.64
Residual unexplained variability							
Proportional error (%CV)	0.139	8.02	12.79	0.137	0.11	0.16	−1.44
Additive error (ng/mL)	2.88	19.81	12.79	2.9035	1.88	4.72	0.82

RES: relative standard error; CL: total clearance (L/h); V_1_: volume of distribution for the central compartment (L); Q: intercompartmental clearance (L/h); V_2_: volume of distribution for the peripheral compartment (L); HNF, on Q: influence of Hourly net fluid volume infused (HNF) on Q; ω_CL_, ω_V1_, ω_Q_ and ω_v2_: the estimates of between-subject variability of CL, V_1_, Q and V_2_, respectively.

All relative precision of the final fixed-effect parameter estimates were less than 30%, and that of the final random-effect parameter estimates were less than 50%. All shrinkages of BSV and RUV except BSV on Q (30.72%) were less than 20%. These suggested that the parameter estimates were reliable. It was worth noting that HNF as a covariate of Q has led to a decrease in BSV from 35.5% to 17.9%, indicating that 17.6% of BSV in Q was explained by HNF.

### 3.3 Model evaluation

**FIGURE 4:**
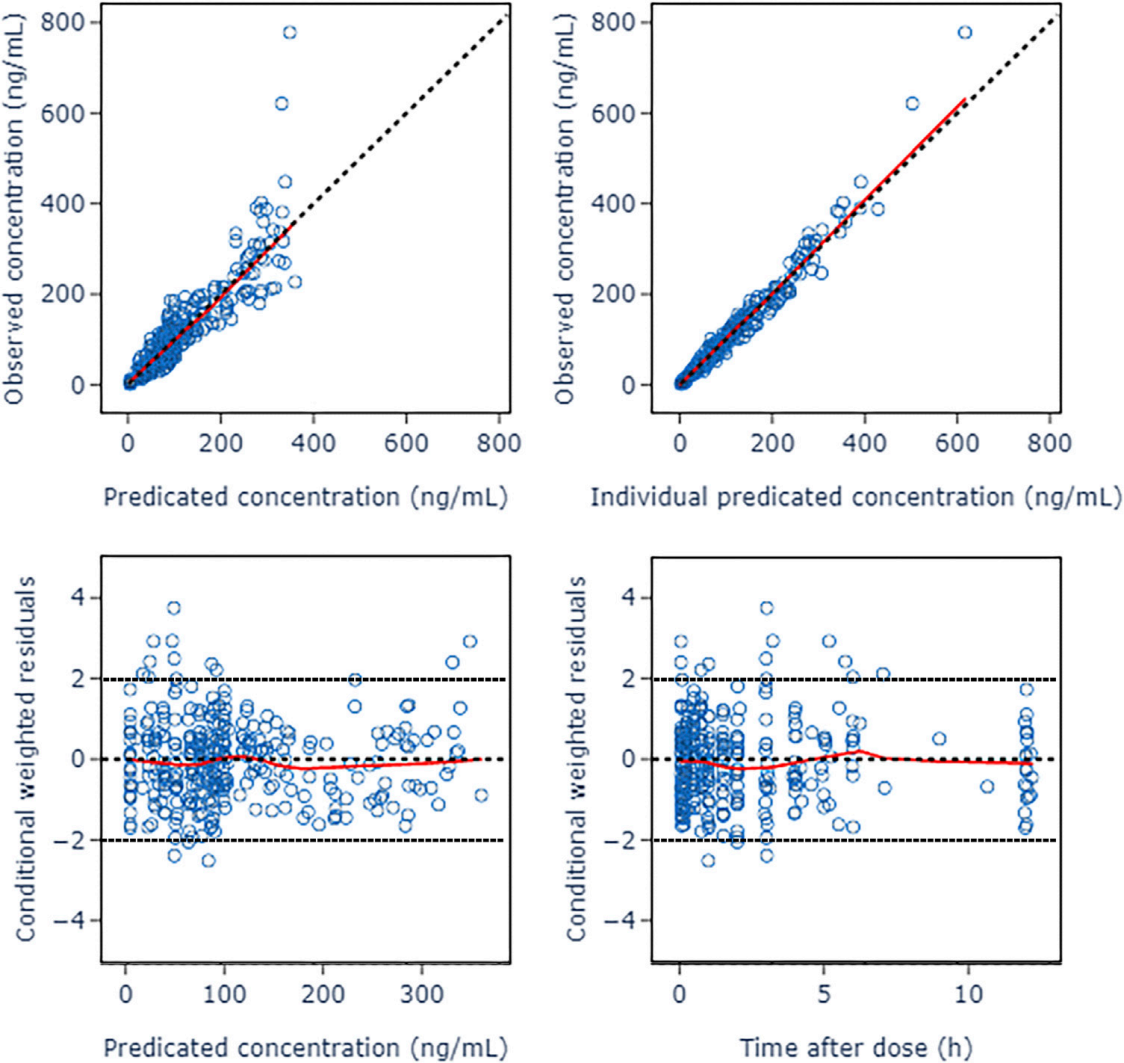
Goodness of fit plots of nalbuphine final PopPK model. The red lines represent the locally weighted scatterplot with smoothing.

The GOF diagnostic plots of the final model was presented in Figure 4. Compared to the basic model, the fitting of IPRED vs. DV and PRED vs. DV plots were improved slightly. The CWRES in the final model was randomly distributed near CWRES = 0 and most of the CWRES were within ± 2, and did not show obvious trends with time, suggesting that the final model well described the trend of data concentration.

The bootstrap with 1,000 times resampling was used to verify the final model, and 984 times of them were successfully minimized. The biases between the final model parameter estimates and the bootstrapped median values were less than 8%. And all of the final model parameter estimates were within 95% confidence interval of the bootstrapped values (Table 4).

**FIGURE 5:**
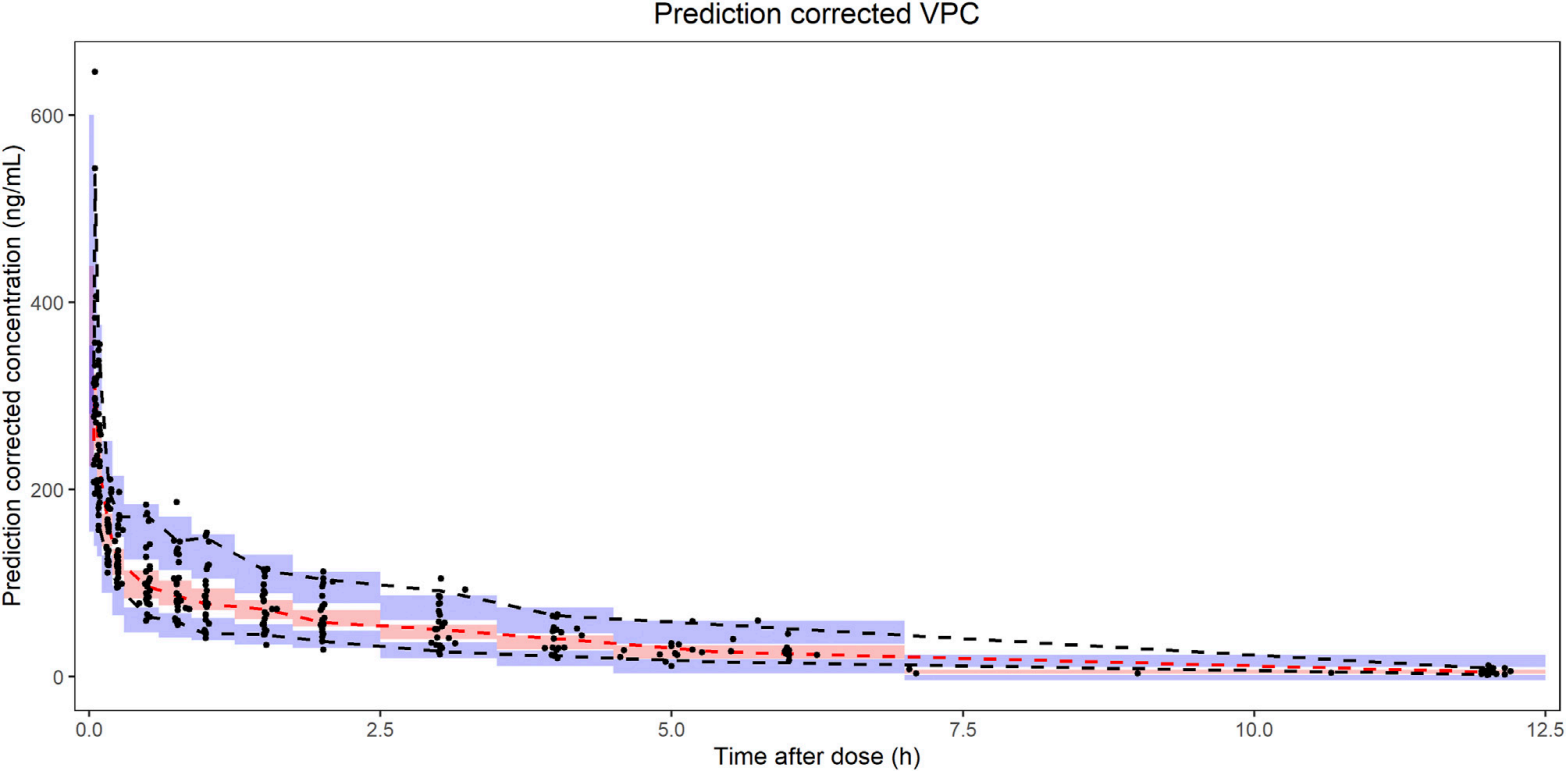
Prediction corrected visual predictive checks (pc-VPC) of nalbuphine final PopPK model. The black dots represented prediction corrected observations. The black dashed lines represented 90% interval of the prediction corrected observations. The red dashed lines represented median of the prediction corrected observations. The red shaded area represented 95% confidence interval (CI) of the median prediction. The blue shaded area represented 95% CI of the 5th and 95th prediction interval.

The pc-VPC plots was presented in Figure 5. The median, 5th and 95th percentiles of the prediction corrected observed nalbuphine concentrations largely overlapped with the 95% confidence interval of the corresponding prediction corrected predicted values from the simulation data. The final model could reasonably describe the observed data.

**FIGURE 6:**
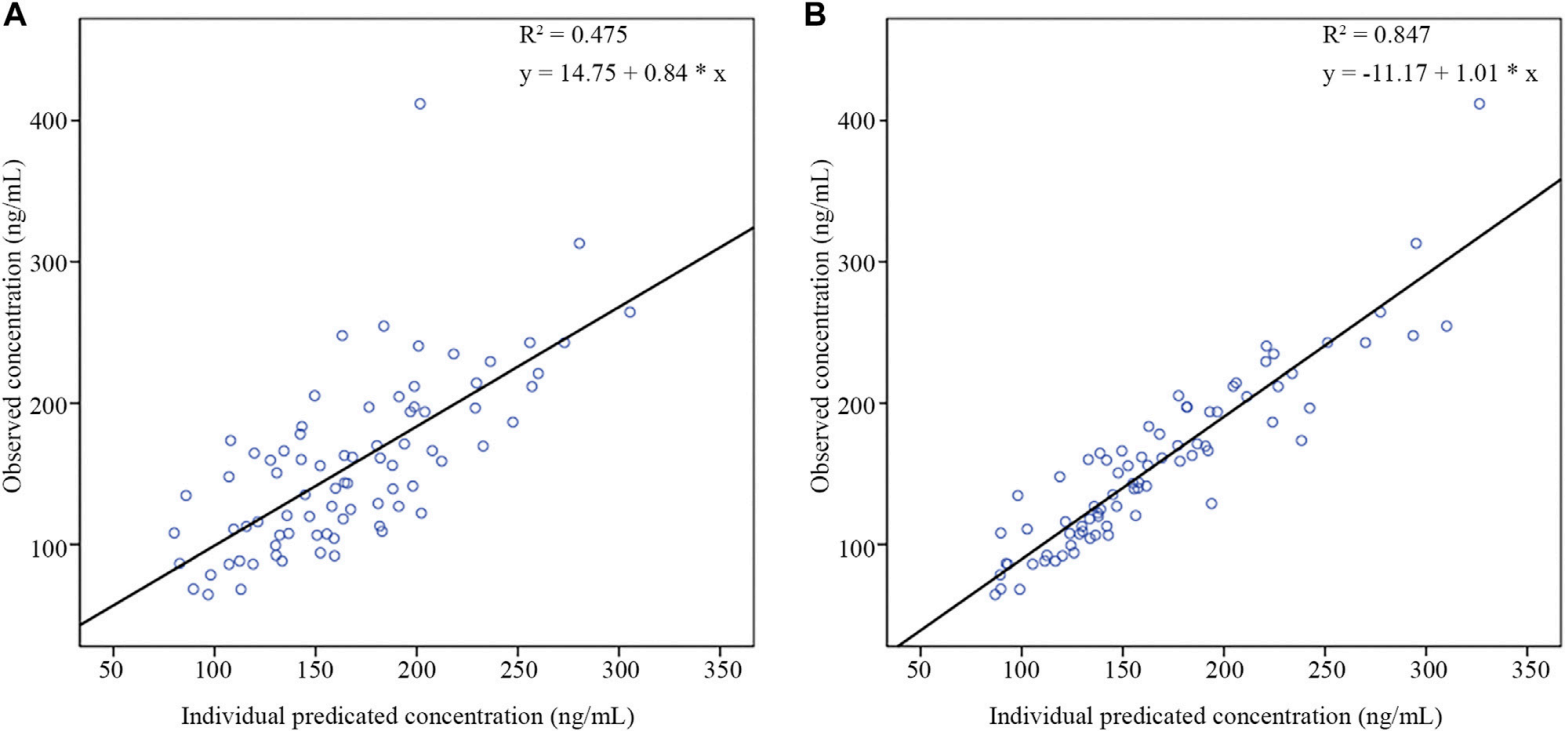
The scatter plots of external validation of the final nalbuphine model. **(A)** external evaluation dataset without observations; **(B)** external evaluation dataset within the first observation of each subject.

As shown in Table 5, the results of external validation of the final model in both scenarios met pre-defined criteria. Compared with validating using datasets with no observations, adding the first observation of each patient to the external validation dataset could reduce MDPE and MAPE by 4.05% and 9.72%, respectively, and increase F_20_ and F_30_ by 28.57% and 22.62%, respectively. The scatterplot of observed versus individual predicted concentrations of two scenarios were presented in Figure 6. All these results showed that the final model sufficiently described the PK of nalbuphine.

**TABLE 5:** External validate results of nalbuphine PopPK model.

Indices	Data without observations	Data within an observation
MDPE (%)	12.97	8.92
MAPE (%)	24.05	14.33
F_20_	40.48	69.05
F_30_	61.90	84.52

PE: prediction error of dependent variable; MDPE: median prediction errors; MAPE: median absolute prediction errors; F_20_, F_30_: PE% within ± 20% and 30%, respectively.

### 3.4 Simulation results

#### 3.4.1 The evaluation of the effect of covariates on the PK of nalbuphine

**FIGURE 7:**
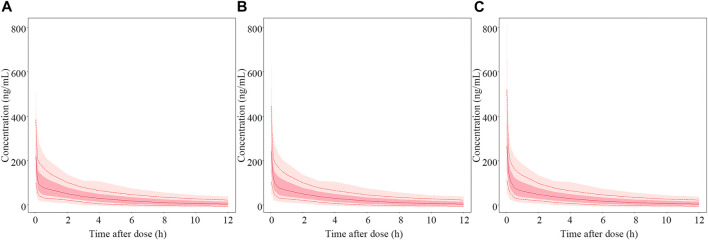
Simulated PK profiles of nalbuphine. The median hourly net fluid volume infused (HNF) of patient in the model building group was 563.6 mL/h. **(A–C)** represent PK profiles of nalbuphine at the 10th, 50th and 90th percentile of HNF levels, respectively. The solid lines represent the median values of 1,000 simulations. The whole shadow and dark shadow represent all simulations and 5% to 95% simulations of the corresponding population in each panel, respectively.

According to the final PopPK model, HNF was the only significant covariate that had an effect on the PK of nalbuphine. Figure 7 showed the predicted concentration profiles for patient populations who were in the 10th, 50th, and 90th percentiles of HNF of the model building population (350.1, 563.6 and 973.4 mL/h, respectively). The simulation results revealed that HNF had limited impact on plasma concentration of nalbuphine.

#### 3.4.2 Dosage regimen simulation and statistical analysis

**FIGURE 8:**
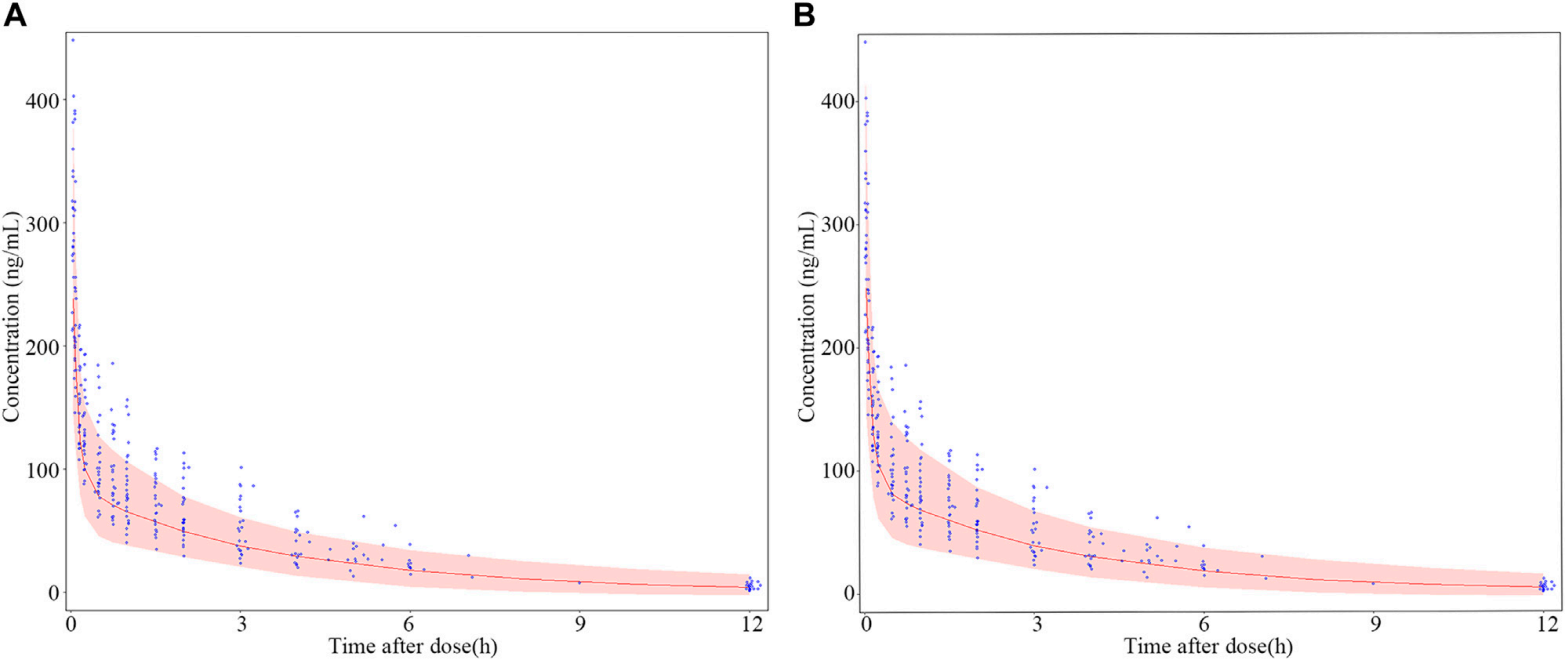
Dosage regimen simulation plots of nalbuphine. **(A)** fixed dosing regimen (12 mg); **(B)** bodyweight dosing regimen (0.2 mg/kg). The blue points were the observed concentration. The red lines represent the 50th percentiles of simulated concentrations distribution in each panel. The shadow represent 5th and 95th percentiles of simulated concentrations distributed in each panel. Two observed concentration (621 μg/mL and 778.1 μg/mL) at 0.05 h were not shown in this Figure.

The results of dosage regimen simulation were presented in Table 6. For two dosage regimens, the bias of C_min_ (0.05 h), C_median_ (4 h) and C_max_ (12 h) means were less than 6%. However, the scatterplot of the predicted concentrations versus time (0–12 h) of two dosage regimens suggested that the PK variability of the fixed dosage regimen was lower than that of the bodyweight dosage regimen (Figure 8).

**TABLE 6:** Statistical analysis results of nalbuphine simulation on two dosage regimens.

Time(h)	Dosage regimen simulation	Mean	Median	Min	Max	Percentile values		Bias (%)
						25th	75th	
0.05	fixed dosing	246.75	241.84	67.76	572.04	203.51	284.82	5.46
	bodyweight dosing	260.22	251.36	62.11	700.00	206.57	306.13	
4	fixed dosing	30.14	29.65	0.46	84.77	23.50	36.19	5.58
	bodyweight dosing	31.82	30.78	0.47	96.07	23.87	38.56	
12	fixed dosing	5.06	3.99	0.00	32.90	1.71	7.39	5.55
	bodyweight dosing	5.34	4.15	0.00	36.73	1.76	7.71	

### 3.5 The effectiveness and safety outcomes of patients

In the course of the trial, only one patient had mild cough during tracheal intubation; all patients were hemodynamically stable with good sedation levels and BIS between 40 and 65 during the operation; all patients recovered consciousness rapidly and did not become agitated during the postoperative awakening period; no patient had postoperative adverse effects such as nausea and vomiting.

## 4 Discussion

This study investigated the PopPK of nalbuphine in adult patients undergoing general anesthesia surgery, and analyzed the effect of a fixed dosage regimen and a body weight dosage regimen on nalbuphine distribution and metabolism.

A two-compartment model got the best description for PK profile of nalbuphine. It was consistent with the model structure of most previous studies (Jaillon et al., 1989; Bressolle et al., 2011; Pfiffner et al., 2022), except for that reported by Jacqz-Aigrain et al. (2003). The typical values of nalbuphine PK parameters from the final model were 32.9 L/h for CL, 32.5 L for V1, 245 L/h for Q and 83.5 L for V2. The results were consistent with our previous results, which were obtained using non-compartmental analysis (NCA) (Gao et al., 2022). While Bressolle et al. (2011) built a PopPK model of nalbuphine after surgery in children (1–11 years) using an allometric scaling model, and their population parameter estimates were 130 L/h/70 kg for CL, 210 L/70 kgforV1, 75.6 L/70 kg for Q and 151 L/70 kg for V2. It revealed that it may be limited to apply the model established with allometric scaling in the children population to the adult population due to the differences in the physiological status of children and adults. The PopPK model developed in this study using data from clinically adult patients could characterize the PK profile of nalbuphine in adults more accurately, especially for patients undergoing general anesthesia surgery. However, compared to the results obtained in healthy adults, the metabolism and distribution of nalbuphine were also significantly decreased in the population in this study. Jaillon et al. (1989) found that the CL, V1, and V2 of nalbuphine in young healthy volunteers (62–90 kg) were 1.783 L/h/kg, 1.97 L/kg and 5.45 L/kg, respectively. He et al. (2021) found that CL and V_d_ of nalbuphine were 90.0 L/h and 326.5 L in healthy adults (55.2–64.2 kg) after 10 mg intravenous injection. As a result, we assumed that variations in the included population and the scenarios of nalbuphine administration were to blame for the disparities in parameters between our research.

A previous study by Sear et al. (1987) found that nalbuphine presented lower clearance (65.7 L/h) and apparent distribution volume (207.6 L) in patients undergoing general anesthesia than in conscious volunteers. There were many factors that might affect the distribution and metabolism of drugs during the operation, and it was difficult to quantify with a single influencing factor (Choi et al., 2017). Large amounts of fluid and blood transfusions during surgery could increase the volume of the systemic circulation, thereby increasing hepatic blood flow and facilitating the distribution of drugs (Eleveld et al., 2014). Conversely, anesthetic drugs and cryogenic environment could reduce the patient’s heart rate and blood pressure, and slow the systemic circulation and the distribution of drugs (Mccollum and Dundee, 1986; Stowe et al., 1992). Zausig et al. (2009) found a 38% decrease in myocardial contractility after intravenous propofol. In addition, surgical stress may also cause a range of hormonal and metabolic changes (Choi et al., 2017). Therefore, the duration of surgery and intraoperative fluid volume, blood transfusion, urine volume, and HNF were chosen to be tested as factors related to surgery during the covariate screening process. And the results found that OFV was significantly reduced (*p* < 0.005) and the model was improved after introducing HNF values as a covariate for Q.

As the hepatic extraction ratio of nalbuphine was estimated to be 0.5–0.7, the influence of HNF on Q of nalbuphine revealed in the present study could be partially explained by the changes of hepatic blood flow (Jaillon et al., 1989; Bressolle et al., 2011). During surgery, fluid and blood transfusions could increase the volume of the patient’s body circulation, which in turn increases hepatic blood flow. The HNF combined with information on volume of fluid infused, volume of blood transfused, volume of urine and duration of operation could reflect the degree of increase in volume of body circulation and hepatic blood flow per unit time of patients. Unexpectedly, the results in the simulations showed that the effect of HNF appeared to be limited. Therefore, clinical pre-adjustment of nalbuphine administration dose based on the level of HNF is not recommended. However, close attention should be paid to the anesthetic effect and hemodynamic changes in patients who might be or had been intraoperatively transfused with higher volumes of fluids, blood transfusions, or blood loss.

Liver is an important organ for drug metabolism and its function can directly affect the PK of drugs. Usually, the livers of adults have fully developed and have a better ability to metabolize drugs compared to infants, children and the elderly. In addition, adult patients with liver disease belong to a special population whose metabolism of drugs is different from that of healthy adults. Hepatic insufficiency could alter the drug distribution and metabolic processes by affecting drug metabolizing enzyme activity, hepatic blood flow and drug binding to plasma proteins (Gao et al., 2022). Nalbuphine is mainly metabolized in the liver mainly by cytochrome P450 enzymes (CYP450) 3A4, 2D6, 2C19 and uridine diphosphate-glucuronyl transferase (UGT) 1A3, 2B7, and clearance is mainly dependent on hepatic blood flow (Bressolle et al., 2011). Our previous NCA analysis found that the t_1/2_ of nalbuphine in patients with hepatic insufficiency was prolonged with increased serum total bilirubin (TBIL) levels (Gao et al., 2022). Although 55.6% of the patients in this study had hepatobiliary disease, there were no covariates related to liver function that could be included in the final model. Notably, alanine aminotransferase (ALT) but not TBIL may have had an effect on the CL of nalbuphine in the present study. During the stepwise process, ALT had been added to the model as a covariate of CL by decreasing the model OFV value of 7.39. Unfortunately, ALT was removed from the full model according to the pre-defined criteria in the backward elimination process. We speculate that this may be due to the small sample size of patients included in this study and the fact that most patients with liver disease had ALT within 3 times the normal value and had insignificant decreases in liver function. Therefore, the effect of ALT on nalbuphine CL needs to be further studied in a larger group of patients with liver disease.

Age and weight were considered to be an important factor affecting the PK characteristics of nalbuphine (Bressolle et al., 2011). Studies in infants, healthy volunteers, and elderly patients found that the CL of nalbuphine significantly decreased with age, which was consistent with the previous univariate analysis (Jaillon et al., 1989; Bessard et al., 1997). And allometric growth models had been used to describe the distribution and metabolism of nalbuphine in infants and children (Jacqz-Aigrain et al., 2003; Bressolle et al., 2011).

However, the introduction of age did not have a significant effect on the final model in this study when building the PopPK model. Changes in organ weight and blood flow had been reported to be the main causes of age-related changes in hepatic clearance (Soejima et al., 2022). In present study, only adult patients undergoing general anesthesia were included, with limited effect of age on their liver weight. In addition, the changes of blood flow caused by surgery were greater than that caused by age. That might be the reason why age was not to be related to the CL of nalbuphine in this study.

In this study, fixed dose regimen (15 mg) was explored in patients undergoing anesthesia surgery based on published literature (Cai et al., 2011; He et al., 2021). During the trial, all patients were hemodynamically stable and well sedated intraoperatively, and there were no adverse effects such as irritability, nausea and vomiting in the postoperative period. Only one patient developed a mild cough during tracheal intubation. Fixed dose of nalbuphine (15 mg) in patients undergoing general anesthesia showed good efficacy and safety. However, 0.2 mg/kg dose regimen was recommended in package inserts of nalbuphine. In order to investigate the effect of body weight on the PK of nalbuphine, the established PopPK model was used to simulate the plasma concentration of nalbuphine in patients with fixed dosage regimen and bodyweight dosage regimen, respectively. Not surprisingly, the bias of the two dosage regimens met the predefined criteria (±15%) either at the beginning of dosage or at the end of the operation period or at the end stage of elimination. However, individualized dosing based on body weight did not reduce inter-patient variability in PK exposure. And fixed dosage regimen is easier to operationalize than weight dosage regimen in clinical, especially for those patients who are critically ill and unable to measure weight. Thus, we would recommend the replacement of bodyweight dosage regimen with fixed dosage regimen in adult patients undergoing general anesthesia surgery.

Several limitations in this study should be considered. First, interactions associated with concomitant drugs were not considered in modeling the PopPK of nalbuphine. Patients in our study received co-administration of nalbuphine, midazolam, sufentanil, and sevoflurane, all of which were metabolized by CYP3A4. However, considering the design of this study, it was unable to identify whether there are PK effects between nalbuphine and other medications used during operation. Second, the investigation of the effect of cancer disease on the PK of nalbuphine was arbitrary. The present study only classified whether the patient had cancer or not. More representative tumor-related indicators need to be identified and included in future analyses of nalbuphine PopPK models. Moreover, there might be some unclear biases in this PopPK model of nalbuphine due to the small size of the sample. It should be verified by more patient data in the future.

## 5 Conclusion

In conclusion, a PopPK model of nalbuphine for adult patients undergoing general anesthesia surgery was developed in this study. While the Q of nalbuphine was significantly affected by the patient’s HNF during surgery, the magnitude of the effect was limited, and no dosage adjustments were recommended. The body weight dosage regimen can be replaced by the fixed drug dosage regimen with low PK variability based on the final PopPK simulation.

## Data Availability

The raw data supporting the conclusion of this article will be made available by the authors, without undue reservation.

## References

[B1] Bessard G. Alibeu J. P. Cartal M. Nicolle E. Serre D. F. Devillier P. (1997). Pharmacokinetics of intrarectal nalbuphine in children undergoing general anaesthesia. Fundam. Clin. Pharmacol. 11 (2), 133–137. 10.1111/j.1472-8206.1997.tb00180.x 9107559

[B2] Bressolle F. Khier S. Rochette A. Kinowski J. M. Dadure C. Capdevila X. (2011). Population pharmacokinetics of nalbuphine after surgery in children. Br. J. Anaesth. 106 (4), 558–565. 10.1093/bja/aer001 21310722

[B3] Cai L. J. Zhang J. Wang X. M. Zhu R. H. Yang J. Zhang Q. Z. (2011). Validated LC-MS/MS assay for the quantitative determination of nalbuphine in human plasma and its application to a pharmacokinetic study. Biomed. Chromatogr. 25 (12), 1308–1314. 10.1002/bmc.1601 21337353

[B4] Chawda P. M. Pareek M. K. Mehta K. D. (2010). Effect of nalbuphine on haemodynamic response to orotracheal intubation. J. Anaesth. Clin. Pharm. 26 (4), 458–460. 10.4103/0970-9185.74584 PMC3087255 21547169

[B5] Choi B. Lee Y. An S. Lee S. Lee E. Noh G. (2017). Population pharmacokinetics and analgesic potency of oxycodone. Br. J. Clin. Pharmacol. 83 (2), 314–325. 10.1111/bcp.13101 27558774 PMC5237696

[B6] Dalens B. J. Pinard A. M. Letourneau D. R. Albert N. T. Truchon R. J. (2006). Prevention of emergence agitation after sevoflurane anesthesia for pediatric cerebral magnetic resonance imaging by small doses of ketamine or nalbuphine administered just before discontinuing anesthesia. Anesth. Analg. 102 (4), 1056–1061. 10.1213/01.ane.0000200282.38041.1f 16551898

[B7] Eleveld D. J. Proost J. H. Cortínez L. I. Absalom A. R. Struys M. M. R. F. (2014). A general purpose pharmacokinetic model for propofol. Anesth. Analgesia 118 (6), 1221–1237. 10.1213/ANE.0000000000000165 24722258

[B8] Errick J. K. Heel R. C. (1983). Nalbuphine. A preliminary review of its pharmacological properties and therapeutic efficacy. Drugs 26 (3), 191–211. 10.2165/00003495-198326030-00002 6137354

[B9] Gao X. N. Nie X. Y. Gao J. L. Heng T. F. Zhang Y. Q. Hua L. (2022). Pharmacokinetic study of nalbuphine in surgical patients undergoing general anesthesia with varying degrees of liver dysfunction. Drug Des. Devel Ther. 16, 2383–2393. 10.2147/DDDT.S371596 PMC9341258 35923933

[B10] Giannina G. Guzman E. R. Lai Y. L. Lake M. F. Cernadas M. Vintzileos A. M. (1995). Comparison of the effects of meperidine and nalbuphine on intrapartum fetal heart rate tracings. Obstet. Gynecol. 86 (3), 441–445. 10.1016/0029-7844(95)00164-M 7651658

[B11] He K. Ji W. Zhao H. Wei Y. Yang S. Wen Q. (2021). Pharmacokinetic comparison of nalbuphine with single injection and patient‐controlled analgesia mimic method in healthy Chinese volunteers. J. Clin. Pharm. Ther. 46 (4), 1166–1172. 10.1111/jcpt.13421 33942343

[B12] Hirsch J. Depalma G. Tsai T. T. Sands L. P. Leung J. M. (2015). Impact of intraoperative hypotension and blood pressure fluctuations on early postoperative delirium after non-cardiac surgery. Br. J. Anaesth. 115 (3), 418–426. 10.1093/bja/aeu458 25616677 PMC4533731

[B13] Jacqz-Aigrain E. Debillon T. Daoud P. Boithias C. Hamon I. Rayet I. (2003). Population pharmacokinetics of nalbuphine in neonates. Paediatr. Perinat. Drug Ther. 5 (4), 190–198. 10.1185/146300903774115793

[B14] Jaillon P. Gardin M. E. Lecocq B. Richard M. O. Meignan S. Blondel Y. (1989). Pharmacokinetics of nalbuphine in infants, young healthy volunteers, and elderly patients. Clin. Pharmacol. Ther. 46 (2), 226–233. 10.1038/clpt.1989.130 2758732

[B15] Kubica-Cielinska A. Zielinska M. (2015). The use of nalbuphine in paediatric anaesthesia. Anaesth. Intensive Ther. 47 (3), 252–256. 10.5603/AIT.2015.0036 26165241

[B16] Mccollum J. S. Dundee J. W. (1986). Comparison of induction characteristics of four intravenous anaesthetic agents. Anaesthesia 41 (10), 995–1000. 10.1111/j.1365-2044.1986.tb12740.x 3491551

[B17] Nguyen T. H. Mouksassi M. S. Holford N. Al-Huniti N. Freedman I. Hooker A. C. (2017). Model evaluation of continuous data pharmacometric models: Metrics and graphics. CPT-PHARMACOMET. Syst. Pharmacol. 6 (2), 87–109. 10.1002/psp4.12161 PMC5321813 27884052

[B18] Pfiffner M. Berger-Olah E. Vonbach P. Pfister M. Gotta V. (2022). Pharmacometric analysis of intranasal and intravenous nalbuphine to optimize pain management in infants. Front. Pediatr. 10, 837492. 10.3389/fped.2022.837492 35311056 PMC8926166

[B19] Schmidt W. K. Tam S. W. Shotzberger G. S. Smith D. J. Clark R. Vernier V. G. (1985). Nalbuphine. Drug Alcohol Depend. 14 (3-4), 339–362. 10.1016/0376-8716(85)90066-3 2986929

[B20] Sear J. W. Keegan M. Kay B. (1987). Disposition of nalbuphine in patients undergoing general anaesthesia. Br. J. Anaesth. 59 (5), 572–575. 10.1093/bja/59.5.572 3580238

[B21] Shuying L. Ping L. Juan N. Dong L. (2016). Different interventions in preventing opioid-induced cough: A meta-analysis. J. Clin. Anesth. 34, 440–447. 10.1016/j.jclinane.2016.05.034 27687431

[B22] Soejima K. Sato H. Hisaka A. (2022). Age-related change in hepatic clearance inferred from multiple population pharmacokinetic studies: Comparison with renal clearance and their associations with organ weight and blood flow. Clin. Pharmacokinet. 61 (2), 295–305. 10.1007/s40262-021-01069-z 34514537

[B23] Stowe D. F. Bosnjak Z. J. Kampine J. P. (1992). Comparison of etomidate, ketamine, midazolam, propofol, and thiopental on function and metabolism of isolated hearts. Anesth. Analg. 74 (4), 547–558. 10.1213/00000539-199204000-00015 1554122

[B24] Wang H. Wang T. Hu X. Deng C. Jiang J. Qin H. (2022). Fixed dosing of kukoamine B in sepsis patients: Results from population pharmacokinetic modelling and simulation. Br. J. Clin. Pharmacol. 88 (9), 4111–4120. 10.1111/bcp.15342 35373389

[B25] Weiser T. G. Regenbogen S. E. Thompson K. D. Haynes A. B. Lipsitz S. R. Berry W. R. (2008). An estimation of the global volume of surgery: A modelling strategy based on available data. Lancet 372 (9633), 139–144. 10.1016/S0140-6736(08)60878-8 18582931

[B26] Zausig Y. A. Busse H. Lunz D. Sinner B. Zink W. Graf B. M. (2009). Cardiac effects of induction agents in the septic rat heart. Crit. Care*.* 13 (5), R144. 10.1186/cc8038 19737388 PMC2784361

